# Prevalence, genetic, and biochemical evaluation of immune response of police dogs infected with *Babesia vogeli*

**DOI:** 10.14202/vetworld.2021.903-912

**Published:** 2021-04-15

**Authors:** Ahmed Adel Zaki, Marwa Mohamed Attia, Elshaimaa Ismael, Olfat Anter Mahdy

**Affiliations:** 1 Veterinary Health Care Unit at k9-Departmeant of Police Academy, First settlement at Cairo, Egypt; 2 Department of Parasitology, Faculty of Veterinary Medicine, Cairo University, 12211 Giza, Egypt; 3 Department of Veterinary Hygiene and Management, Faculty of Veterinary Medicine, Cairo University, Giza 12211, Egypt

**Keywords:** *Babesia vogeli*, Egypt, *Ehrlichia canis*, police dogs, tumor necrosis factor-alpha

## Abstract

**Background and Aim:**

*Babesia* species are tick-borne protozoan parasites of apicomplexan type which infect the erythrocytes of dogs it ranges from subclinical to severe cases, depending on different factors such as immune status, age, and presence of other co-infections with the *Babesia* species. Hence, this study aimed to identify the protozoan parasites infecting police dogs of different breeds, ages, and both sexes in Egypt. Concerning molecular detection of *Babesia vogeli* using conventional polymerase chain reaction sequencing and phylogenetic analysis, followed by the assessment of immunological and biochemical status of infected dogs.

**Materials and Methods:**

The blood of 242 police K9 dogs was collected. The age, breed, sex, and health status with clinical signs of dogs were recorded. Hematological, biochemical, and oxidative stress analyses of the blood were performed together with gene expression analysis using two genes (gamma interferon [IFN-γ] and tumor necrosis factor-alpha [TNF-α]). The identification of the causative agent was performed using molecular analysis of the 18S ribosomal RNA (rRNA). The 18S rRNA region of canine *Babesia* spp. was successfully amplified, and sequencing data were deposited in GenBank (accession number: MT565474.1), which resembled those of *B. vogeli*.

**Results:**

The results of blood samples screening revealed that of the 242 blood samples, 62 were positive for *B. vogeli* infection. The infection rate in male dogs was higher than that in female dogs. The police dogs were classified into the following three groups of dogs: (1^st^ group) healthy, (2^nd^ infected with *B. vogeli*, and mixed infection of *B. vogeli* and *Ehrlichia canis*). The oxidative stress biomarkers levels in *B. vogeli* infected dogs were greater than that of healthy dogs. Likewise, IFN-γ and TNF-α level in *B. vogeli* infected dogs were elevated in infected dogs.

**Conclusion:**

Our findings demonstrated that *B. vogeli* had completely adverse effects on the health condition of the police dogs that may lead to death in some dogs.

## Introduction

Dogs have several roles, for example, being a guard dog and in explosive and narcotic detection. These dogs may have several diseases such as bacterial, viral, and parasitic infections. Tick infection is one of the most common diseases in dogs; there are several protozoan parasites that infect these dogs, including *Babesia canis*, *Hepatozoon canis*, and *Ehrlichia canis* [1]. Babesiosis and ehrlichiosis are the most common, especially in summer. The infected dogs experience thrombocytopenia that characterizing babesiosis and hyperglobulinemia that characterizing ehrlichiosis [2,3]. The clinical signs of babesiosis in dogs include fever, hemolysis of red blood cells with anemia, hyperbilirubinemia, hemoglobinuria, jaundice, and organ failure [4,5] *B. canis* are tick-borne protozoan parasites of apicomplexan type that infect the erythrocytes of dogs [6-8]. Babesiosis ranges from subclinical to severe cases, depending on different factors such as immune status, age, and presence of other co-infections with the *Babesia* spp. [9,10]. The diagnosis of babesiosis depends on the microscopic detection of the intraerythrocytic piroplasms in peripheral blood smears [10,11].

The significance of this study as mentioned in a recent study by Badawi and Yousif [12] provides the molecular record and phylogenetic analysis of *B. canis* in dogs in Iraq, and it will be valuable to confirm clinical signs and study epidemiological risk factors of babesiosis in dogs. However, various species from the genus *Babesia* (*B. canis*, *Babesia vogeli*, and *Babesia rossi*) cannot be differentiated through microscopic examination of blood films, despite differences in their genetics, pathology, and vector associations [13].

Therefore, this study aimed to identify the protozoan parasites infecting police dogs of different breeds, ages, and both sexes in Egypt by interrupting the molecular detection of *B. vogeli* using conventional polymerase chain reaction (PCR) sequencing and phylogenetic analyses, followed by the assessment of immunological and biochemical status of infected dogs.

## Materials and Methods

### Ethical approval

Institutional and National guidelines for the care and use of animals were followed as directed by Faculty of Veterinary Medicine, Cairo University.

### Study period and location

The study was conducted from August 2017 to December 2019. Samples were collected at Veterinary Health Care Unit at K9-Department of Police Academy and processed at Biotechnology Laboratory, Cairo University Research Park.

### Sample collection

A total of 242 blood samples were collected from police dogs at (K9-department) in police Academy located in the first settlement in Cairo, Egypt. The survey was conducted along various seasons, from August (Summer), October (Autumn), December and February (Winter), as well as April and June (Spring). The ages of the dogs ranged between 2.5 and 12 years. General inspection indicated that 180 police dogs were healthy without visible clinical signs. However, 62 dogs exhibited clinical manifestations such as fever, emaciation, anemia, nostril and anal bleeding, edema, jaundice, and bloody urine. Many ways of control were used to eradicate and prevent further infection and infestation of ticks and protozoal parasites using tick repellents and antiprotozoal drugs. The detailed information, such as sex, breed, age, and health status of these dogs as well as season, is presented in Table-1. Across all samples, the percentage of female dogs was 16.12% (39 of 242), whereas the percentage of male dogs was 83.88% (203 of 242).

**Table-1:** Prevalence of *Babesia* in Police dogs from Egypt (n=242).

Variables	No. of tested dogs (%) (n=242)	*Babesia* infected dogs (n=62/242)		Mixed infection with Ehrlichia (n=28/62)	
	
		No. (%)	CI 95%	No. (%)	CI 95%
Breeds:					
German shepherd	182 (75.21)	51 (28.02)	21.5-34.6	24 (47.06)	33.4-60.8
Malinois	52 (21.49)	10 (19.23)	8.5-29.9	4 (40.00)	9.6-70.4
Dutch Shepherd	7 (2.89)	0.0	0.0	0.0	0.0
Belgian shepherd	1 (0.41)	1 (100)	100	0.0	0.0
p-value		0.071		0.858	
Gender:					
Female	39 (16.12)	8 (20.51)	7.8-33.2	4 (50.00)	15.4-84.7
Male	203 (83.88)	54 (26.60)	20.5-32.7	24 (44.44)	31.2-57.7
p-value		0.425		1.000	
Intensity of tick infection:					
No	108 (44.63)	0.0	0.0	0.0	0.0
Mild	37 (15.29)	0.0	0.0	0.0	0.0
Moderate	53 (21.90)	24 (45.28)b	31.9-58.7	10 (41.67)	21.9-61.4
Severe	44 (18.18)	38 (86.36)a	76.2-96.5	18 (47.37)	31.5-63.2
p-value		< 0.0001		0.660	
Age (years):					
≤3	62 (25.62)	9 (14.52)	0.0-32.2	1 (11.11)b	0.0-31.6
4-6	86 (35.54)	26 (30.23)	20.5-39.9	12 (46.15)ab	26.9-65.3
7-9	83 (34.30)	25 (30.12)	20.3-39.9	15 (60.00)a	40.8-79.2
≥10	11 (4.55)	2 (18.18)	0.0-40.9	0.0	0.0
p-value		0.103		0.035	
Health status:					
Normal	180 (66.12)	0.0b	0.0	0.0	0.0
Clinical signs	62 (33.88)	62 (75.61)a	66.3-84.9	27 (43.55)	31.2-55.9
P-value		<0.0001			
Status:					
Live	182 (75.21)	9 (3.72)b	1.8-8.1	1 (11.11)	0.0-31.6
Died	60 (24.79)	53 (88.33)a	80.2-96.5	26 (49.06)	35.6-62.5
p-value		<0.0001		0.065	
Season:					
Autumn	37 (15.29)	21 (56.76)a	40.8-72.7	11 (52.38)	31.0-73.7
Winter	72 (29.75)	26 (36.11)b	25.0-47.2	14 (53.85)	34.7-73.0
Spring	41 (16.94)	6 (14.63)c	3.8-25.5	1 (16.67)	0.0-61.7
Summer	92 (38.02)	9 (9.78) c	3.7-15.9	1 (11.11)	0.0-31.6
p-value		<0.0001		0.060	

a,b,c Different superscripts within the same column indicate significance at P*<*0.05; CI 95%=Confidence interval 95%

### Collection of blood samples

Blood samples were collected from the cephalic vein of 242 police dogs; each sample was divided into two parts: One with ethylenediaminetetraacetic acid (EDTA) as an anticoagulant for preparing the examination of blood films and DNA extraction and the second one without an anticoagulant for serum separation for biochemical analysis.

### Parasitological examination

The blood smears were placed on a clean slide, air dried, fixed in absolute methyl alcohol for 10 min, and Giemsa stained as per the manufacturer’s instruction. The smears were washed with tap water to remove extra stains and then air-dried. The stained blood smears were examined by light microscopy (×40 and ×100) (OLYMPUS CX41, Japan) for the detection of blood parasites.

### Hematological analysis of blood infected with B. canis

A total of 180 whole blood samples with EDTA were subjected into hematological analysis to determine different blood parameters that could be changed during babesiosis infection in dogs. In addition, sera were subjected to biochemical analysis to determine aspartate aminotransferase (AST), alanine aminotransferase (ALT), and C-reactive protein (CRP) using specific test kits (Spectrum Diagnostics, Egypt) [14,15].

### PCR analysis of the blood for identification of the *B. canis* subspecies

Molecular analysis of frozen blood from infested dogs with babesiosis was subjected to DNA extraction. The DNA was extracted from 200 μL of blood using the DNA blood kit (QIAGEN, Hilden, Germany) in the automatic extraction system QIAcube (QIAGEN). In each round of extraction, one sample of DNase/RNase-Free distilled water was included as a blind control for DNA extraction. To detect members of the genera *Babesia*, a fragment (~560 bp) of the 18S ribosomal RNA (rRNA) gene was amplified and sequenced using the forward primer PIRO-A (5′- AATACCCAATCCTGACACAGGG-3′) and PIRO-B (5′-TTAAATACGAAT GCCCCCAAC-3′) that amplify complete 18S rRNA gene under conditions described by Olmeda *et al*. [16]. The PCR cycling was: Initial denaturation for 2 min at 95°C for denaturation, annealing 30 s at 55°C, and 30 s at 72°C for extension and a finally 5 min at 72°C for extension. The resulting sequences were assembled using the SeqMan Pro software, edited with Edit Seq tools in Lasergene (DNASTAR, Inc., Madison, WI, USA) and compared with available sequences using Basic Local Alignment Search Tool (BLAST) in GenBank.

### Measurement of oxidative stress markers

Different parameters of oxidative stress were evaluated in sera. The levels of malondialdehyde (MDA), superoxide dismutase (SOD), and catalase were evaluated using the positive and negative sera with the specific kit analysis [17,18].

### Evaluation of tumor necrosis factor-alpha (TNF-α) and gamma interferon (IFN-γ) activity

Blood samples from the infected dogs had *B. vogeli* with blood films, and blood samples from five young reared uninfected puppies were collected in a similar manner and used as negative controls. All blood samples were aseptically preserved in −20°C.

#### RNA isolation

RNA isolation from 100 mg of blood was performed by a total RNA kit (Ambion, Applied Biosystems, USA) per the manufacturer’s instructions. The Thermo Scientific Nano-Drop was used to measure the RNA purity and quantity. Notably, 500 ng of RNA was made with DNase I amplification grade (Invitrogen, Germany) per the manufacturer’s instructions. The reverse transcription of treated RNA was performed by High-Capacity cDNA Archive Kit (Applied Biosystems) [19].

#### Quantitative real-time PCR protocol

PCR primer set specific for TNF-α and IFN-γ specific for dogs was designed and based on sequences deposited in the GenBank. β-actin was used as a reference gene and for sample normalization. The gene expression included in this study was tested on a separate pool of cDNA, generated from five uninfected dogs previously examined for the presence of any parasites [20].

### Statistical analysis

The PASW Statistics version 18.0 software (SPSS Inc., Chicago, IL, USA) was used for data analysis. The Chi-square (*χ*^2^) test or Fisher’s exact test (FET) was used to determine the differences between *Babesia* infection rates under various parameters. Hematological, biochemical, oxidative stress, and gene expression parameters of healthy and diseased dogs were compared by one-way analysis of variance and independent sample t-test and expressed as mean±standard error. p<0.05 [21] was considered statistically significant.

## Results

### Morphological identification of the protozoan parasite

**Figure-1:**
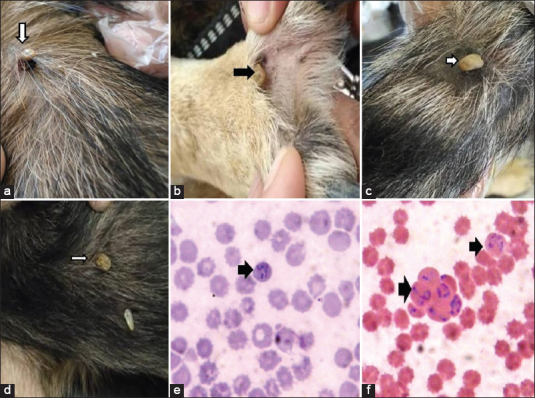
*Ticks* (*Rhipicephalus sanguineus* which identified by light microscope infection in Police dogs (A-D), E: F: stained blood smears revealed presence of *Babesia vogeli* with large form (3-5.5 μm in length). Each piroplasma was pyriform in shape, pointed at one end, and round at other end using 100×.

The infected dogs experienced tick infection from the genus *Rhipicephalus sanguineus* (Figure-1) which was identified by a light microscope. The examined stained blood smears revealed the presence of *B. canis* with a large form (3-5.5 μm in length). Each piroplasma was pyriform in shape, pointed at one end and round at the other end (Figure-1).

### Molecular identification of *B. canis* infected police dogs

The 18S rRNA region of canine *Babesia* spp. was successfully amplified using the universal primers mentioned earlier in this study. Purified PCR products from this blood protozoan parasite was directly sequenced and yielded 554 bp of three specimens. These sequences were deposited in GenBank (accession number: MT565474.1). The comparison of these DNA sequences fragments with other nucleotide sequences and the divergence of *B. canis canis* and *B. vogeli* in GenBank indicated that this species was identified and confirmed as *B. vogeli*.

The BLAST analysis of *B. vogeli* (MT565474.1) of this study revealed 100.0% nucleotide identity with *B. vogeli* (AY371197.1 in Egypt and HQ662635.1 in Romania), 99.61% nucleotide similarity with *B. vogeli* (KT333456.1 in Brazil and KM199636.1 in Thailand), 96.41% nucleotide similarity with *B. vogeli* (MK674799.1 in France), 99.21% similarity (MT740272.1) in Kenya and HQ148663 in Taiwan, and 98.62% and 98.52% nucleotide similarity with *B. vogeli* (DQ439545.1 and AY150061.1 in Spain). In contrast, the present nucleotide similarity with *B. canis canis* was 96.08% nucleotide similarity (KX839230.1 in Italy, EU622793.1 in Poland, KF499115.1 in Turkey, HQ662634.1 in Romania, and FJ209025.1 in Croatia).

**Figure-2:**
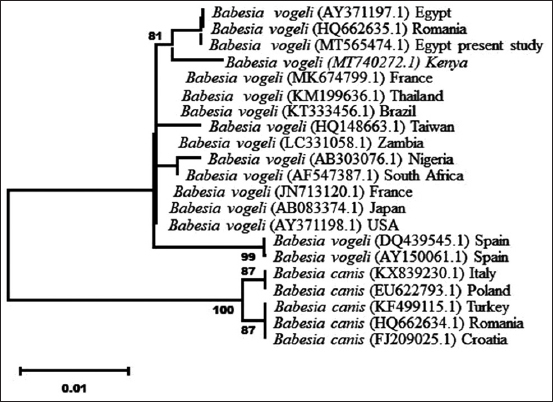
Phylogenetic tree of the 18S rDNA gene sequences created using the neighbor-joining method and bootstrap analysis of 500 replicates. Sequences with accession numbers have been taken from GenBank for comparison. The sample sequenced in the present study represents another identical sequence of the first genetic variant detected in this study (GA combination).

The derived phylogenetic tree based on the neighbor-joining model using the 18S rRNA region of *B. vogeli* revealed Two strong nodal supports of *B. canis* major clades. The first clade included *B. canis* grouped with *B. vogeli* and the second clade included *B. canis canis* to form two subclades. The first subclade of *B. vogeli* grouped Egypt and Romania together and separated from other groups of *B. canis canis* (Figure-2).

### Prevalence study of *B. vogeli* in police dogs

The results of blood samples screening revealed that of the 242 blood samples, 62 were positive for *B. vogeli* infection (25.62% [62 of 242]; 95% confidence interval [CI], 20.12-31.12) (Table-1). No significant association was observed between the breed of dogs and *B. vogeli* infection rates (p=0.071; FET). The infection rate in male dogs (26.60% [54 of 203]; 95% CI, 20.52-32.68) was higher than in female dogs (20.51% [8 of 39]; 95% CI, 7.84-33.19); however, the difference was not significant (*χ*^2^ [1, N=242] = 0.636; p=0.425). Meanwhile, no significant association was observed among the various ages of dogs (*χ*^2^ [3, N=242] = 0.617; p=0.103). Notably, the infection rate in dogs that were severely infected with ticks was higher than those with lower rates of infection (*χ*^2^ [3, N=242] = 145.90; p<0.0001), and dogs showing clinical signs presented a significantly higher infection rate than the apparently normal dogs (*χ*^2^ [1, N=242] = 162.65; p<0.0001). Similarly, the detection rate of *Babesia* in dead dogs was significantly higher than in living dogs (*χ*^2^ [1, N=242] = 164.66; p<0.0001). Furthermore, the infection rates in autumn and winter were significantly higher than that in spring and summer (*χ*^2^ [3, N=242] = 37.69; p<0.0001).

### Detection of mixed infection with *B. vogeli* and *E. canis*

The results of blood samples screening revealed that of the 62 positive *Babesia* blood samples, 28 were positive for *E. canis* infection (45.16% [28 of 62]; 95% CI, 32.77-57.55) (Table-1). No significant association was observed between the breed of dogs (p=0.858; FET), sex (p=0.530; FET), alive or dead status (p=0.065; FET), intensity of tick infection (*χ*^2^ [1, N=62] = 0.193; p=0.660), season of the year (p=0.060; FET), and rates of mixed *Babesia* and *E. canis* infection. Notably, the rates of mixed *Babesia* and *E. canis* infection in older dogs were significantly higher than in younger ones (p=0.035; FET).

### Hematological and biochemical analysis

Hematological and biochemical results of sampled police dogs were classified into three groups, that is, healthy dogs, dogs infected with *B. vogeli*, and dogs that acquired mixed infection of *Babesia* and *E. canis* (Table-2). The hematology results of the sampled police dogs are presented in Table-2. Infected dogs exhibited lower hemoglobin concentration, packed cell volume (PCV), and total erythrocyte count than healthy dogs. Most of the dogs with low hematological values also clinically manifested anemia. Notably, low platelet count and leukopenia were observed in dogs with mixed *Babesia* and *E. canis* infection.

**Table-2:** Hematological and biochemical parameters in healthy and infected dogs (n=180).

Parameters(normal/reference range)	Mean±SE			*F*(2, 177)	p-value

	Healthy dogs (n=127)	*Babesia*infected dogs (n=27)	Mixed *Babesia*and *Ehrlichia* infection (n=26)		
Hematology:					
Total erythrocyte count(5.58.5×106μl)	6.92±0.08a	4.44±0.15b	4.43±0.23b	142.19	<0.0001*
Hemoglobin concentration(1218g%)	14.33±0.13a	8.97±0.49b	9.56±0.29b	177.06	<0.0001
Platelet count(200900×102μl)	264.21±6.40a	240.44±14.75a	58.12±14.24b	86.56	<0.0001
Packed cell volume(Hematocrit)(3755%)	40.44±0.52a	35.04±0.27b	34.44±0.47b	24.26	<0.0001
Total leukocyte count(617×106μl)	10.12±0.26a	10.27±0.52a	4.36±0.34b	49.88	<0.0001
Biochemical:					
AST(848 IU/L)	40.46±1.35b	94.15±6.15a	84.38±5.11a	110.28	<0.0001
ALT(858 IU/L)	51.39±1.62b	120.74±5.46a	117.42±4.86a	193.93	<0.0001
Creatinine(0.51.6 mg%)	0.91±0.17c	1.89±0.08b	2.20±0.08a	323.65	<0.0001
Blood urea nitrogen (8.826 mg%)	26.13±0.37c	55.44±3.36b	66.46±3.07a	253.19	<0.0001

a,b,cDifferent superscripts within the same row indicate significant difference at P*<*0.05and confidence level 95%; SE=Standard error, AST=Aspartate aminotransferase, ALT=Alanine aminotransferase. *F* (2, 177): *F*-value(degree of freedoms between groups, degree of freedoms within groups).

* When the statistical test gives a P result=0.000, we express it as<0.0001

The biochemical results of the serum samples obtained from police dogs are presented in Table-2. High AST and ALT levels were observed in both dogs infected with *Babesia* and dogs that acquired mixed *B. vogeli* and *E. canis* infection, which indicated liver damage. Symptoms of liver damage clinically manifested as edema and jaundice. Increased levels of creatinine and blood urea nitrogen were observed in dogs infected with *Babesia* and dogs that acquired mixed *B. vogeli* and *E. canis* infection, which indicated renal failure. Symptoms of renal failure clinically manifested as emaciation and bloody urine.

### Oxidative stress

**Figure-3:**
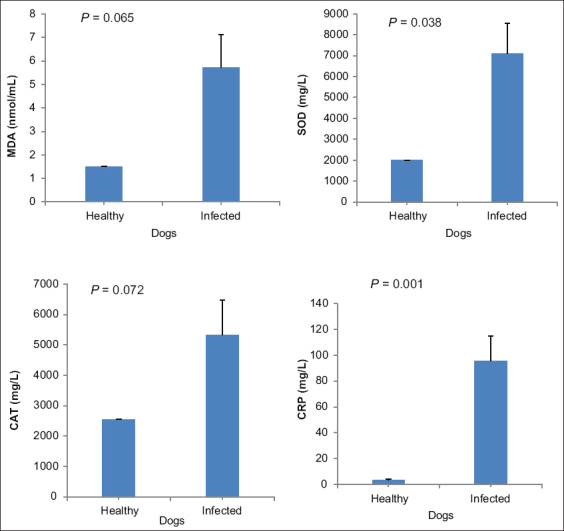
MDA=Malondialdehyde; SOD=Superoxide dismutase; CAT=Catalase and CRP (C-reactive protein). Significance was indicated at p<0.05.

On average, MDA levels in *B. vogeli* infected dogs (5.72±1.41) were greater than that of healthy dogs (1.50±0.01). This mean difference (4.22 nmol/mL [95% CI, ±4.49]) was not significant (*t* [*df*=6] = 2.25; p=0.065). Similarly, CAT levels in *Babesia* infected dogs (5326.80±1139.70) were greater than that in healthy dogs (2554.00±0.58). This mean difference (2772.80 nmol/mL [95% CI, ±3164.33]) was not significant (*t* [4] = 2.43; p=0.072]). However, SOD levels in *Babesia* infected dogs (7101.40±1439.28) were greater than that in healthy dogs (1997.67±1.33). This mean difference (5103.73 nmol/mL [95% CI, ±4695.71]) was significant (*t* [6] = 2.66; p=0.038]). Moreover, CRP levels in *Babesia* infected dogs (95.85±18.88) were greater than that in healthy dogs (3.50±0.38). This mean difference (92.35 [95% CI, ±42.71]) was significant (*t* [6] = 4.89; p=0.001]) (Table-3, Figure-3).

**Table-3:** Oxidative stress and gene expression parameters in healthy and infected dogs.

Oxidative stress and gene expression parameters	Healthy dogs	*Babesia*infected dogs	*t(df*)	p-value
MDA(nmol/mL)	1.50±0.00	5.72±1.41	–2.25(6)	0.065
SOD(mg/L)	1997.67±1.33b	7101.40±1439.28a	–2.66(6)	0.038
CAT(mg/L)	2554.00±0.58	5326.80±1139.70	–2.43(4)	0.072
CRP(mg/L)	3.50±0.38b	95.85±18.88a	–4.89(9)	0.001
IFNγ(U/mL)	3.33±0.17b	8.20±1.16a	–3.14(6)	0.02
TNFα(U/mL)	2.97±0.03b	10.60±1.96a	–2.91(6)	0.027

a,bDifferent superscripts within the same row indicate significant difference at p<0.05. MDA=Malondialdehyde, SOD=Superoxide dismutase, CAT=Catalase, CRP=C-reactive protein, IFN-γ=Gamma interferon, TNF-α=Tumor necrosis factor-alpha

### Gene expression analysis

**Figure-4:**
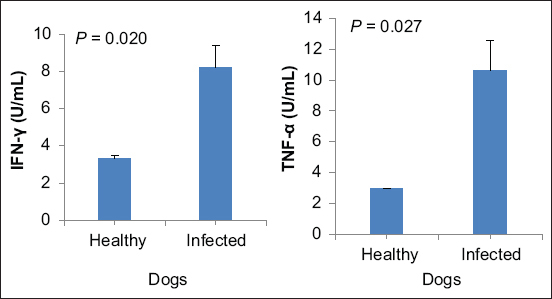
IFN-γ=Gamma-interferon; TNF-α=Tumor necrosis factor alpha. Significance was indicated at p<0.05.

Likewise, IFN-γ levels in *Babesia* infected dogs (8.20±1.16) were greater than that in healthy dogs (3.33±0.17). This difference (4.87 [95% CI, −8.66 to −1.08]) was significant (t [6] = −3.14; p=0.020]). In addition, TNF-α levels in *Babesia* infected dogs (10.60±1.96) were greater than that in healthy dogs (2.97±0.03). This difference (7.63 U/mL [95% CI, −14.04 to −1.22]) was significant (t [6] = −2.91; p=0.027]) (Table-3, Figure-4).

## Discussion

In this study, we investigated the prevalence of 2 tick-borne pathogens in police working dogs in Egypt. Our results indicated the prevalence of *B. vogeli* (25.62%) and *E. canis* (11.57%). The previous studies reported that the prevalence of *Babesia* spp. in dogs ranged from 1.2% to 11.3% in China [22,23], 10.9% to 19.09% in India [24], 14.0% in Southern Italy [25], and 61.1% in Nigeria [26]. Results showed that breed, sex, and age did not significantly influence the incidence of *B. vogeli*, which agreed with. Adebayo *et al*. [26]. Canine babesiosis did not take enough consideration in research work in Egypt compared with other animal species [27]. Other studies investigated that the prevalence of *E. canis* in dogs was 10.3% in Korea [28], 1.3% in Southern China [29], 44.6% in North Trinidad [30], 29.26% in Mexico [31], and 40.6% in Colombia [32]. Although our study contradicts with reports on age difference regarding positivity to *E. canis*, there was a significant increase in *E. canis* infection rate in older dogs (7 years of age and older), which suggests that acquiring infection increases with age [30]. The diverse patterns for canine tick-borne pathogens among different countries may be attributed to the differences in the tick distribution [28].

The current study noted that the rate of *Babesia* infection increased by age (>3 years) and declined after the age of 9 years; however, the difference was not significant. This result is consistent with Salem and Farag [27] who reported that the highest infections were found in the age group of 3-5 years and explained that *Babesia* infection was known to increase by age, reaching its peak between the age of 3 and 5 years and then peter out [33]. Similarly, older years of age emerged as a risk factor for *B. canis*/*B. vogeli* sero-reactivity as suggested in the study by Veneziano *et al*. [25]. They stated that this finding is probably caused by the cumulative exposure to the relevant tick vectors rather than an increased susceptibility to the *Babesia* infection. However, other previous studies indicated that although canine babesiosis can occur in animals of all ages, young animals are more susceptible to *Babesia* infection and exhibited more severe clinical manifestations [23].

Furthermore, the current study remarked non-significant higher rates of infection in male dogs than females, which agrees with Mellanby *et al*. and Veneziano *et al*. [25,34] who stated that male dogs exhibited an increased risk of developing canine babesiosis, because they have a higher environmental exposure due to increased roaming behavior. Salem and Farag [27] stated that male dogs exhibited a higher infection rate than female ones; the aggressiveness and hormonal status of male dogs may be a contributory factor here. However, Martinod *et al*. [35] found no difference in sex susceptibility between males and females.

The study indicated that the rate of *Babesia* infection was not dependent on the breed of the surveyed dogs. However, earlier studies indicated a significantly higher seroprevalence in long-hair dogs, in which hard ticks can easily attach and avoid detection [25]. In addition, a breed predisposition has been suggested in Hungary, citing the vulnerability of the German shepherd breed to developing babesiosis caused by *B. canis* [33]. Salem and Farag [27] reported that German shepherd dogs seemed to be the breed with the highest infection rate, which was attributed to the prevalence of this breed in Egypt, which agreed with our findings.

Results reported that the rate of *B. canis* infection was significantly dependent on the intensity of tick infection in surveyed dogs. This finding comes in agreement with Farag [36] who reported that the tick population plays a crucial role in the incidence of the infection. Furthermore, the rate of *Babesia* infection was significantly dependent on the season of the year. The prevalence of *Babesia* infections was significantly higher in cold months (autumn and winter), which agrees with Farag [36] who stated that climatic condition represents a notable factor in the incidence of the infection. In addition, Veneziano *et al*. [25] stated that the tick vector favors cool and wet weather.

*Babesia* infections were significantly associated with the manifestation of clinical signs. The main clinical signs observed were fever, anemia, jaundice, edema, and bleeding. Schetters *et al*. [37] described the classical presentation of *Babesia* infections as febrile illness with apparent anemia. Most of the dead dog cases (88.33%) were positive for *Babesia*; 49.06% of them acquired co-infection with *E. canis*. Adebayo *et al*. [26] reported that babesiosis accounts for approximately 40% mortality in the canine cases presented in hospitals in Africa.

In the current study, we reported co-infection with *E. canis* in 28 of 62 dogs (45.16%) infected with *Babesia*. This study reported a significantly higher rate of *B. vogeli* and *E. canis* co-infection in old dogs than young ones. Co-infection with other pathogens, such as *E. canis*, is common in endemic areas, depending on the geographic location and the distribution of the arthropod vectors. In addition, co-infection is of major clinical relevance, because it complicates diagnoses, intensifies clinical symptoms, diminishes the effectiveness of the medication, and could worsen the prognosis [38,39].

Anemia was the most hematologic alterations observed in *B. vogeli* infected dogs. The mechanisms of inducing anemia have been poorly investigated. Salem and Farag [27] attributed the cause of anemia to the destruction of circulating red blood cells by autoantibodies. Leisewitz *et al*. [13] stated that the high incidence of hemoglobinuria is testimony to the very important role hemolysis plays in anemia. Thrombocytopenia, anemia, and leukopenia were the most hematologic alterations observed in the dogs that acquired co-infection with *E. canis*. Thrombocytopenia is the most consistent hematological finding detected in dogs infested with *E. canis* as reported by Frank and Breitschwerdt [40]. These findings agreed with the study by Asgarali *et al*. [30] who reported that 80.4% of *E. canis* seropositive dogs had low platelet counts. Moreover, Niwetpathomwat *et al*. [41] reported severe anemia in 18%, leukopenia in 19%, and thrombocytopenia in 93% of the dogs studied. Thrombocytopenia was attributed to multiple causes, such as increased platelet consumption, destruction and sequestration of platelets in the spleen, and co-infection with *Babesia* [42,43].

The significantly elevated levels of ALT and AST observed in *B. vogeli* and *E. canis* infected dogs were frequently reported in the previous studies and indicate the possibility of liver injury after primary infection [44,45]. However, Zygner *et al*. [44] noted that elevated AST levels can also be associated with kidney injury. All blood biochemical indicators of kidney function were significantly higher in *B. canis* infected dogs than healthy controls. Zygner *et al*. [46] in his study on dogs naturally infected with *Babesia* stated that creatinine was significantly correlated with urea concentration. However, they stated that high creatinine level is considered a poor prognostic indicator.

In our study of the canine babesiosis, there is a correlative association between TNF-α concentration, clinical severity, and parasitemia Zygner *et al*. [47]. TNF-α and IFN-γ play a significant role in the production of several harmful substances such as nitric oxide and free oxygen radicals, which could initiate the interaction between parasitized erythrocytes and the blood vessel wall [48]. Different metabolites of the nitric substance were considered as one of the mediators of multiple organ dysfunction syndromes that are present in canine babesiosis.

This study found a great difference in CRP between healthy and babesiosis-infested canine blood, whereas a study on *B. rossi* infections showed no differences in CRP levels between survivors and non-survivors [15]. However, an analysis showed that CRP was significantly associated with glucose levels. In dogs with *Babesia gibsoni*, CRP has a positive correlation with PCV recovery; hence, it could be used as a biomarker of disease condition and clearance [14]. This confirms the idea that the inflammatory mechanisms in canine babesiosis are important to be identified because tissue hypoxia and metabolic dysfunction play a great process in the disease process and condition.

Babesiosis infection was based on a diagnosis made by the morphology of stages of *Babesia* species within blood cells, but the similarity between species and subspecies was a restricting agent [49]. The PCR method represents a powerful tool for determining not only the genotype but also in which cases symptoms appear nonspecific and/or the blood smears do not present a particular thing the diagnosis. There are recent advances in molecular methodology (methods based on PCR and subsequent DNA sequencing). Genetic characterization was possible to detect and identify piroplasms with greater sensitivity and specificity than conventional ones [50]. Consequently, the knowledge on the prevalence of *Babesia* species and subspecies infecting police dogs has increased significantly.

Characterization by molecular methods confirmed the presence of three distinct genotypes of *B. vogeli*. Among these, *B. vogeli* was recognized as the most common agent of canine babesiosis in temperate regions of Europe [51]. In this study, the detected *B. vogeli* agreed with the results recorded by Elsafey [52], Farag [36], and Abdel-Rhman *et al*. [53] who reported that *B. vogeli* was found to naturally infect dogs in Egypt.

Compared with the 18S rRNA sequence obtained through the present BLAST analysis of *B. vogeli* (GenBank accession number: MT565474.1) with other *Babesia* species, these sequences were completely identical to a *B. vogeli* sequence from Egypt and Romania (AY371197.1 [54] and HQ662635.1 [55], respectively).

The present results indicate a very close genetic relation with 99.61% nucleotide similarity and the affiliation of the canine *Babesia* from Brazil (KT333456.1) [56] to *B. vogeli*. When the present sequences were compared with the sequence of the canine *Babesia* from *B. vogeli*, 96.41% nucleotide similarity was found in France (MK674799.1) [57], whereas 99.21% similarity was found in Kenya (MT740272.1) [58] and in Taiwan (HQ148663.1) [59]. In contrast, the present nucleotide similarity of *B. vogeli* was compared with *B. canis canis*; 96.08% nucleotide similarity was found in Italy (KX839230.1) [60], in Poland (EU622793.1) [61], in Turkey (KF499115.1) [62], in Romania (HQ662634.1) [55], and in Croatia (FJ209025.1).

## Conclusion

The causative agent of tick-borne illness in police dogs was *B. vogeli*. Therefore, from this study, the dogs must be treated against the babesiosis and tick infection together with spraying of the area where the dogs live (ground, dog houses, and walls). Breed disposition, sex, and age did not significantly influence the incidence of *B. vogeli*. The potential contribution of tick exposure, sex, or breed predisposition requires further epidemiological studies.

## Authors’ Contributions

AAZ: Collection of the samples. OAM and MMA identified the parasites and did the serological analysis EI: Did the statistical analysis. All authors sharing in writing this manuscript and revised it. All authors have read and approved the final manuscript.
